# Sum of the Magnitude for Hard Decision Decoding Algorithm Based on Loop Update Detection

**DOI:** 10.3390/s18010236

**Published:** 2018-01-15

**Authors:** Jiahui Meng, Danfeng Zhao, Hai Tian, Liang Zhang

**Affiliations:** College of Information & Communication Engineering, Harbin Engineering University, Harbin 150001, China; zdfeng@hrbeu.edu.cn (D.Z.); tianhai123@hrbeu.edu.cn (H.T.); zhangliang@hrbeu.edu.cn (L.Z.)

**Keywords:** 5G, non-binary LDPC, hard decision decoding, reliability, loop detection, sum of magnitude

## Abstract

In order to improve the performance of non-binary low-density parity check codes (LDPC) hard decision decoding algorithm and to reduce the complexity of decoding, a sum of the magnitude for hard decision decoding algorithm based on loop update detection is proposed. This will also ensure the reliability, stability and high transmission rate of 5G mobile communication. The algorithm is based on the hard decision decoding algorithm (HDA) and uses the soft information from the channel to calculate the reliability, while the sum of the variable nodes’ (VN) magnitude is excluded for computing the reliability of the parity checks. At the same time, the reliability information of the variable node is considered and the loop update detection algorithm is introduced. The bit corresponding to the error code word is flipped multiple times, before this is searched in the order of most likely error probability to finally find the correct code word. Simulation results show that the performance of one of the improved schemes is better than the weighted symbol flipping (WSF) algorithm under different hexadecimal numbers by about 2.2 dB and 2.35 dB at the bit error rate (BER) of 10^−5^ over an additive white Gaussian noise (AWGN) channel, respectively. Furthermore, the average number of decoding iterations is significantly reduced.

## 1. Introduction

On 14 October 2016, the 3rd generation partnership project radio layer 1 (3GPP RAN1) conference determined the long-code block coding scheme for 5G communication using the low density parity check node (LDPC) code as the data information of the mobile bandwidth enhance mobile broadband (eMBB) service [1,2]. The 5G includes both people-centric and machine-centric communications [3,4]. These two types of scenarios have different needs. People-centric communications pursue high performance and high-speed communications. Corresponding end-user data rates should reach 10 Gbps and base station data rates should reach 1 Tbps. Machine-centric communication pursues low power consumption with the corresponding sensor rate of only 10–100 Bps, while industrial control applications require a particularly high delay of 10^−4^ s [5].

Gallager proposed the LDPC code [6]. Furthermore, he created the bit flip hard decision decoding algorithm based on the check and statistics as well as proposing the soft decision decoding algorithm based on a posterior probability (APP). The decoding algorithms of multiple LDPC codes are divided into two categories according to the different decision methods [7]: Soft decision algorithm (SDA) and hard decision algorithm (HDA). The representative algorithm of the SDA is the belief propagation (BP) decoding algorithm [8,9]. The error correction performance of the BP algorithm is excellent, but the computational complexity of the BP algorithm is high with a large amount of computation and a large amount of cached data that must be stored in memory during decoding iteration, which is not conducive to engineering implementation [10]. Therefore, the SDA is chosen mainly from the perspective of reducing the decoding complexity of the study [11], with the improved algorithm applied to 5G mobile communications in the human-centric high-speed communications. The representative algorithm in HDA is a bit-flipping (BF) algorithm [12], which has low complexity and computational complexity. The algorithm is simple and easy to implement in hardware, but the decoding performance is not as good as the SDA. Therefore, the HDA is mainly studied from the perspective of improving the decoding performance, with the improved algorithm applied to a machine-centric low-rate, low-latency system in 5G mobile communications.

In the future, the 5G mobile communication system in 2020 will provide higher data transmission rate, more service connections and better user experience while ensuring low cost, transmission security, reliability and stability [13]. Channel coding in 5G mobile communication involves non-binary LDPC codes [14]. Although non-binary LDPC codes have excellent error correction performance, the complexity of high decoding is the main reason that restricts its practical application. Therefore, this paper optimizes the performance of the HDA with low complexity, and optimizes it using the symbol flipping (SF) algorithm. For the hard decision decoding algorithm, the weighting symbol flipping (WSF) algorithm proposed in literature [15] constructs a new flip function by using the minimum value of the variable node amplitude adjacent to the check node (CN) as the reliability of the check equation. On this basis, the reliability information of VN is used as the input of the flip function by the weighting factor and thus, the performance of the modified weighted symbol flipping (MWSF) algorithm will be improved [16]. Based on the belief propagation theory, the decoding algorithm proposed by literature [17] achieves a certain degree of coding gain. In this paper, this is called the improved MWSF (IMWSF) algorithm. Since then, there have been proposals of an average probability and stopping criterion weighted symbol flipping (APSCWSF) algorithm [18,19], multiple-vote symbol flipping (MV-SF) algorithm [20,21,22] and so on [23,24].

Current communication systems need to have faster and more efficient decoders than previous ones. On this basis, many scholars have studied the fast convergence and low complexity decoding algorithm with minimum decoding loss [25,26,27,28]. For the WSF algorithm proposed in literature [19,25], the destruction of the parity relation is attributed to the lowest reliability symbol in the check equation. However, if one check equation is not established, all the symbols involved in the equation may be wrong, but the symbols with lower reliability are more likely to be wrong. Therefore, a new calculation method of reliability is redefined in this paper. When calculating the reliability of variable nodes from each check equation, the reliability information of each variable node participating in the check is taken into account. Furthermore, the sum of the amplitudes of the variable nodes adjacent to the check node is used as the reliability of the check equation. At the same time, when calculating the reliability of participating check symbols, excluding the information carried by the symbol itself, a weighted symbol flipping decoding algorithm based on the magnitude sum (SMWSF) is proposed to obtain more effective soft information for reliability. On this basis, the calculation method of flip function is improved, because the variable node’s own reliability information plays an important role in the construction of the flip function. The weighted value of the symbol itself is added by a weighting factor and a modified weighted symbol flipping decoding algorithm based on the magnitude sum (MSMWSF) is proposed to improve the efficiency of symbol flipping. This is intended to improve the decoding performance and speed up the convergence. As there is a problem of repeatedly flipping the wrong symbols when the symbol is flipped, a new detection algorithm of infinite loops is proposed in this paper. Combined with the MSMWSF algorithm, this paper proposes a sum of the magnitude for weighted symbol flipping decoding algorithm based on loop update detection (LUDMSMWSF) to further speed up the convergence rate and reduce the decoding complexity.

## 2. Basic Definitions

In the non-binary (N,K) LDPC codes, N is the code word length and K is the information bits length. The non-binary LDPC codes are determined by the sparse non-binary parity check matrix ***H***, while the check matrix ***H*** has M≥N−K rows and N columns. Each row represents a check equation and each column represents a code word. For any hard decision vector y, the check equation is $s_{h}^{\left(k\right)}=y^{\left(k\right)}H^{T}$, where the real numbers involved are carried out under the Galois field. h represents the elements in the checksum matrix. k represents the number of iterations. The number of non-zero elements in each row and each column in the check matrix is equal, which is called the rule code, while the others are called the irregular codes. The row weight of the check matrix ***H*** is $d_{c}$ and the column weight is $d_{v}$. A non-“0” element on the nth column of the Galois field is represented by M(n) in the check matrix ***H***. A non-“0” element on the *m*th row of the Galois field is represented by N(m) in the check matrix ***H***. $M\left(n\right):=\left\{m:h_{mn}\neq 0\right\}$, $N\left(m\right):=\left\{n:h_{mn}\neq 0\right\}$.

We selected any code word $c=\left[c_{0},c_{1},\cdots ,c_{N-1}\right]\in {\left\{\mathrm{GF}\left(q\right)\right\}}^{N}$, where $q=2^{b}$. Before sending, the code word is mapped to the bipolar vector $t=\left[t_{0},t_{1},\cdots ,t_{N-1}\right]$ according to the transmitted signal constellation Ω, where $t_{n}=\left[t_{nb},t_{nb+1},\cdots ,t_{\left(n+1\right)b-1}\right],0\leq n<N$. Using Binary Phase Shift Keying (BPSK) modulation, $\phi :\mathrm{GF}\left(q\right)\to \Omega^{b}$, where Ω={−1，1}. The specific mapping rule is $\phi \left(c_{n}\right)=t_{n}=\left[t_{nb},\cdots ,t_{\left(n+1\right)b-1}\right]$, where $t_{i}\in \Omega ,0\leq i<Nb$. After the transmission of the Additive White Gaussian Noise (AWGN), the output is:

(1) r=t+n

where n is the independent white noise real vector with noise power $\sigma^{2}={\left(2R_{c}E_{b}/N_{0}\right)}^{-1}$; $E_{b}/N_{0}$ is the signal-to-noise ratio of each information bit; and $R_{c}=K/N$ is the bit rate.

## 3. Algorithm Description

### 3.1. Sum of the Magnitude of Weighted Symbol Flipping Decoding Algorithm

The WSF decoding algorithm uses the information derived from the channel to calculate the reliability [15,20]. The core idea is to compute the measure of each symbol according to the reliability of the check equation. According to the size of the measured value, the most unreliable symbol is selected to be flipped. It is the simplest SF decoding algorithm based on the hard decision. The flip function $E_{n,\alpha }^{\left(k\right)}$ of the MWSF algorithm and the IMWSF algorithm takes into account the information carried by the symbol itself, but still neglects the reliability information of a large number of variable nodes when calculating the reliability of the check equation $w_{n,m,\alpha }$ [19,25]. However, the variable node involved in each check equation is a set and the reliability calculation of the check equation should also contain the reliability information for each variable node in the set. Therefore, in this paper, the reliability and flip function are redefined. Based on the hard decision decoding, some soft information from the channel is used to calculate the reliability. The sum of the amplitude of the variable nodes connected to the check node is used as the reliability of the check equation, the influence of all symbols is added into the reliability of the check equation and the weighting factor is introduced. Using $\left|L_{n,\alpha }\right|$ as part of the flip function, we obtained the WSF algorithm based on the sum of the magnitude (SMWSF). The specific process of SMWSF decoding algorithm is as follows.

- Step 1: Initialization parameters
  1. Set initial iterations k=0
  2. Calculate the initial hard decision output sequence, before using this sequence as the output of the decoding iteration in k=0, which is denoted as $y^{\left(0\right)}$

Firstly, the hard decision sequence $x=\left[x_{0},x_{1},\cdots ,x_{Nb-1}\right]$ is obtained by the hard decision of the binary sequence r of channel output. The rules of the decision are as follows: If $r_{i}\geq 0$, the $x_{i}$ is determined to be 1; if $r_{i}<0$, the $x_{i}$ is determined to be 0 (0≤i<Nb−1). As the hard decision is a binary sequence, so according to the binary and q-ary conversion principle, converted to N length, the sequence becomes $y=\left[y_{0},y_{1},\cdots ,y_{N-1}\right]$. The sequence of $y=\left[y_{0},y_{1},\cdots ,y_{N-1}\right]$ is the output sequence.

3. Calculate the probability vector $L_{n}\left(\alpha \right)$

After the hard decision, it is necessary to calculate the initial likelihood probability of the symbol and to prepare the average likelihood probability of the check equation adjacent to all variable nodes in the subsequent iterative decoding process. The definition ${\mathrm{GF}}_{0}\left(q\right)=\left\{\alpha_{1},\cdots ,\alpha_{q-1}\right\}$ to remove the Galois field of zero element, while GF(q) does not contain zero elements. Gives the reliability vector α on the nth symbol:

(2) $$L_{n}=\left[L_{n,\alpha_{1}},\cdots ,L_{n,\alpha },\cdots ,L_{n,\alpha_{q-1}}\right]$$

where the symbol probability log likelihood is shown as follows:

(3) $$L_{n,\alpha }=\ln\frac{P\left(a=\alpha_{i}\right)}{P\left(a=0\right)}=\frac{2}{\delta^{2}}\sum_{j:\phi {\left(\alpha \right)}_{j}=+1}r_{nb+j}$$

Obviously, the log likelihood ratio of symbol probability is directly proportional to $\sum_{j:\phi {\left(\alpha \right)}_{j}=+1}r_{nb+j}$. In this present study, we ignored the coefficient $\frac{\delta^{2}}{2}$ related to channel noise [29] and used $\sum_{j:\phi {\left(\alpha \right)}_{j}=+1}r_{nb+j}$ approximation as the symbol probability log likelihood ratio, without any effect on performance.

4. Compute the reliability $w_{n,m,\alpha }$ of the external information of the received symbol $y_{n}$

We defined $L_{n,\alpha }=\sum_{j:\phi {\left(\alpha \right)}_{j}=+1}r_{nb+j}\left(0\leq j\leq b-1\right)$ and used Equation (3) to calculate probability matrix $L_{n}=\left[L_{n,\alpha }\right],0\leq n<N,\alpha_{1}\leq \alpha <\alpha_{q-1}$. In this, $w_{n,m,\alpha }$ represents the average probability information of the symbol α with all the variable nodes adjacent to the *m*th check equation. As shown in Equation (4), the formula of $w_{n,m,\alpha }$ is:

(4) $$w_{n,m,\alpha }=\sum_{i\in N\left(m\right)n}\left|L_{i,\alpha }\right|,0\leq n<N,0\leq m<M,\alpha \in {\mathrm{GF}}_{0}\left(q\right)$$

- Step 2: Calculating check equation $s_{h}^{\left(k\right)}$

We assume that the symbol vector is $y^{\left(k-1\right)}$ before the k(k≥1) iteration, while the corresponding calibration equation is $s_{h}^{\left(k-1\right)}=y^{\left(k-1\right)}H^{T}\neq 0$. Then, the check equation $s_{h}^{\left(k\right)}$ is calculated, $s_{h}^{\left(k\right)}=y^{\left(k\right)}H^{T}$. If the check equation $s_{h}^{\left(k\right)}=0$, the decoding iteration is stopped and the decoding is shown to be successful.

- Step 3: k←k+1, if the $k>k_{\max}$ ($k_{\max}$ is the maximum number of iterations set for the user), the decoding is declared to fail and stop.

The completion of a symbol of the flip need to determine the two parameters: (1) The position of the flip; and (2) the value or amplitude of the flip.

- Step 4: Determining the position of the flip symbol

We calculated the measured value $E_{n}^{\left(k\right)},0\leq n<N$ for each variable node at the k^th^ iteration. The core of the WSF algorithm involves flipping out the symbols that do not satisfy the check equation. To calculate $E_{n}^{\left(k\right)}$, we first need to calculate the reliability of variable node n, respectively $\left\{\alpha_{1},\cdots ,\alpha_{q-1}\right\}$.

(5) $$E_{n,\alpha }^{\left(k\right)}=\sum_{m\in M\left(n\right)}\left(2s_{h,m}^{\left(k\right)}-1\right)w_{n,m,\alpha }-\beta \left|L_{n,\alpha }\right|$$

In Equation (5), β(β≥0) is the weighting factor. At that time, β=0, which allows us to integrate the MSMWSF algorithm into SMWSF algorithm. For the given non-binary LDPC codes, the optimal value of the weighting factor can be obtained by Monte Carlo simulation under the specified number of iterations [30]. 

After this, we update the check equation to get the adjacency value of the hard decision:

(6) $$s_{h,m}^{\left(k\right)}=\left\{ \begin{array}{l} 0,s_{h}^{\left(k\right)}=0\\ 1,s_{h}^{\left(k\right)}\neq 0 \end{array}\right.$$

Among them, m is the CN. If $s_{h}^{\left(k\right)}=0$, a valid code word is obtained and the search is stopped. If $s_{h}^{\left(k\right)}\neq 0$, the flip function $E_{n}^{\left(k+1\right)}$, 0≤n<N is calculated and looped accordingly.

In order to estimate the reliability measure of a symbol, the measure of each symbol must be calculated:

(7) $$E_{n}^{\left(k\right)}=\sum_{\alpha \in {\mathrm{GF}}_{0}\left(q\right)}E_{n,\alpha }^{\left(k\right)}$$

We select the location where the symbol is to be flipped, which is $n^{\left(k\right)},0\leq n<N$.

The summation operation in Equation (7) is a weighted method to measure the location possibility of flipping symbols. The sum of the weights of 1 can enhance the measure of the symbol position and provide a more accurate judgment standard. In this way, the symbol corresponding to the maximum value of $E_{n}^{\left(k\right)}$ is the position of the flip symbol, such as the Equation (8).

(8) $$n^{\left(k\right)}=\arg\max E_{n}^{\left(k\right)},n\in \left[1,N\right],n\notin A$$

- Step 5: Determining the value or magnitude of the flip

For the chosen symbol $y_{n}^{\left(k\right)}$, flip the bit corresponding to the minimum value of $\left|r_{ni}\right|$, where 0≤i<b, and update $y^{\left(k-1\right)}$.

- Step 6: Decoding, according to the results of the flip to get a new hard decision decoding sequence.

Assign the value of $y^{\left(k-1\right)}$ to $y^{\left(k\right)}$, $y^{\left(k\right)}\leftarrow y^{\left(k-1\right)}$, and return to the second step.

### 3.2. Loop Update Detection Algorithm

Due to the possibility of an infinite loop in the process of symbol flipping, the symbol after the current flipping is still an error symbol and cannot be flipped to correct the symbol. Therefore, this paper proposes a loop update detection algorithm to further improve the decoding performance and speed up the convergence.

Firstly, the output symbol sequence matrix and the infinite loop detection matrix are defined, which involves the sequential traversal of each symbol. After this, sorting them from smallest to largest, we determine the location of the flip symbol. From the second smallest symbol, we re-flip the same error symbol, which is converted to the bit value. The wrong bits are sorted from smallest to largest. According to the definition of small to large index value, the bit is flipped and then converted into a symbol value to replace the previous decoding symbol. The symbol position corresponding to the maximum value is placed in the exclusion sequence. Essentially, after the symbol position corresponding to the maximum value is excluded, the maximum value error symbol is searched again. This process is repeated for decoding the iteration. The specific decoding process is as follows.

●Step 1: Initialization

The excluded sequence ***A*** is initialized to an empty set, which is used to store the position corresponding to the symbol that does not satisfy the flipping function. The bit flipping identifier ***F*** is defined and initialized to 1, which is used as the counter. The maximum value is ***b*** (1bq) and the value of ***F*** determines the number of bits to be flipped in the flip symbol.

●Step 2: Determining the magnitude of symbol flipping

The bit position to be flipped in the symbol position $n^{\left(k\right)}$ is determined by the binary sequence $r=\left[r_{0},r_{1},\cdots ,r_{Nb-1}\right]$, which is transmitted after the AWGN channel is transmitted. The symbol at position $n^{\left(k\right)}$ of the symbol in the to-be-flipped state can be converted into b bits, which correspond to $\left[r_{nb},r_{nb+1},\cdots ,r_{\left(n+1\right)b-1}\right]$ in **r**. We sort $\left|r_{i}\right|\left(nb\leq i\leq \left(n+1\right)b-1\right)$ from largest to smallest, with a smaller $\left|r_{i}\right|$ indicating lower reliability of the corresponding bit. Therefore, the ***F*** bit position with the smallest absolute value in $\left[r_{nb},r_{nb+1},\cdots ,r_{\left(n+1\right)b-1}\right]$ is selected according to the bit flipping identifier ***F***, while the F bits in the corresponding position are reversed to obtain a new symbol sequence $y^{\left(k\right)}=\left[y_{0},y_{1},\cdots ,y_{N-1}\right]$.

●Step 3: Detects whether there is an infinite loop

The loop update detection algorithm (LUD) is as follows. First, the output symbol sequence matrix $Y_{\left(k+1\right)\times N}$ is defined, as shown in Equation (9).

(9) $$Y=\left[\begin{array}{c} y^{\left(0\right)}\\ y^{\left(1\right)}\\ \vdots \\ y^{\left(k\right)} \end{array}\right]$$

Following this, an infinite loop detection matrix $E_{k\times N}$ is defined, as shown in Equation (10).

(10) $$E=\left[\begin{array}{c} y^{\left(k\right)}-y^{\left(0\right)}\\ y^{\left(k\right)}-y^{\left(1\right)}\\ \vdots \\ y^{\left(k\right)}-y^{\left(k-1\right)} \end{array}\right]$$

As long as all the elements of one row in an infinite loop matrix are 0, an infinite loop is detected. Otherwise, an infinite loop is not detected.

(1) If an infinite loop is detected and F does not reach the maximum ***b***, we increase the value of ***F*** by 1. After this, we return to step 2 to re-determine the position of the specific bit to be flipped by the symbol, before flipping the ***F*** bits of the corresponding position.
(2) If an infinite loop is detected but ***F*** has reached the maximum ***b***, the currently selected flip symbol position is stored in the exclusion symbol sequence ***A***. ***F*** is set to 1 and the flip symbol position is rediscovered.
(3) If no infinite loop is detected, the exclusion symbol sequence ***A*** is set as an empty set, the bit flipping identifier ***F*** is 1 and the calibration equation is recalculated.

**Figure 1:**
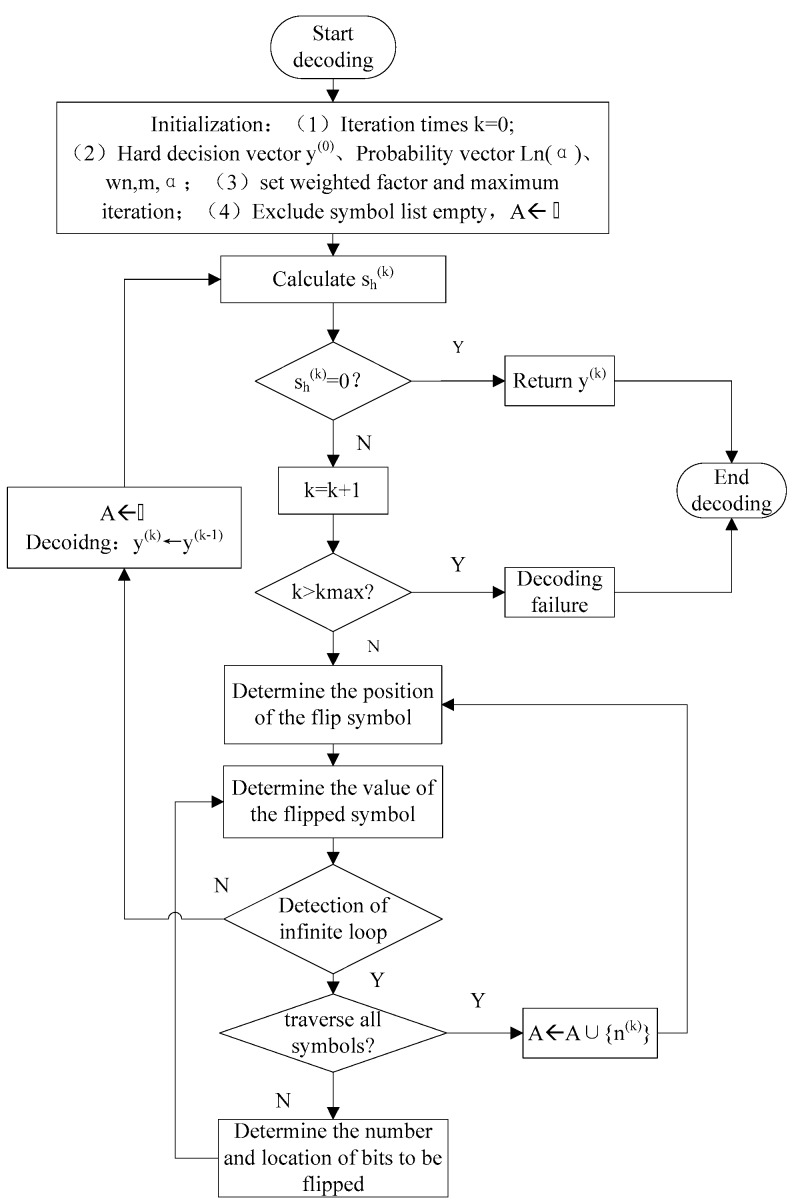
Flow chart of LUDMSMWSF algorithm.

Combining the loop update detection algorithm with the weighted symbol flipping algorithm based on sum of magnitude, a modified sum of the magnitude for the weighted symbol flipping decoding algorithm based on loop update detection (LUDMSMWSF) is proposed. On the basis of the MSMWSF algorithm, the algorithm repeatedly flips the bits corresponding to the error code word and looks for them in the order of the most probable error probability. Finally, this algorithm finds the correct code word. If this error traverses all the symbols, there is still an infinite loop, which indicates that the current symbol is not an error symbol. Following this, the algorithm adds the current symbol to the excluded symbol set to ensure that the next current position will not be continuously found. The specific decoding algorithm flow chart is shown in Figure 1.

## 4. Complexity Analysis

In this section, the conditions used to analyze the complexity of the decoding algorithm are: (1) Ignoring a small number of binary calculation and multiplication operations in the algorithm, with the assumption that the comparison operation is equivalent to the addition operation; and (2) taking the regular non-binary LDPC codes as an example to compare the average number of real addition operations in each algorithm. In the past, the computational complexity of the initial stage before the first iteration is often neglected when analyzing the computational complexity [31]. However, the simulation analysis of the decoding algorithm in the next section shows that the highest signal-to-noise ratio (SNR) in the frame occurs through iterative decoding less frequently after convergence. Thus, there is strong complexity of the initialization phase in the decoding process of the high proportion, if the neglect leads to a significant error. Therefore, the computational complexity involved in the initialization stage before the first iteration is taken into account. At the same time, the principle of second sections shows that all WSF algorithms perform symbol flipping in the final stage of each iteration, which is a serial decoding algorithm. After the symbol flipping is completed, the check equation is updated and the flip function is updated. In each iteration, there are three steps involved: (1) Calculating the adjoining vector $s_{h}^{\left(k\right)}$; (2) updating the flip function $E_{n}^{k+1}$; and (3) searching the flipped bit. Taking the standard WSF algorithm as an example, if the variable node n is flipped during the previous iteration, the adjoining vectors $s_{h}^{\left(k\right)}$ corresponding to the $d_{v}$ check nodes connected to them need to be updated. After the update of the vector $s_{h}^{\left(k\right)}$ is completed, the flip function $E_{n}^{k+1}$ of $d_{c}$ variable nodes connected to the check node m is calculated. Therefore, for both steps, the total number of operations required is $d_{c}\cdot d_{v}$. In Step (3), it is assumed that the node position of the variable corresponding to the maximum flip function $E_{n}^{k+1}$ is found in n variable nodes, while further comparison operations are performed for N−1 times. Therefore, the calculation amount of an iterative process in a standard WSF algorithm is $N-1+d_{c}\cdot d_{v}$. The specific update process is as follows. First, we calculate $s_{h,m}^{k+1}=1-s_{h,m}^{k},m\in M\left(\theta \right)$ and update it according to the updated formula of flip function, which is $E_{n}^{k+1}=E_{n}^{k}+2\sum_{m\in M\left(\theta \right)}w_{n,m,\alpha }\left(2s_{h,m}^{k+1}-1\right),n\in N\left(m\right),m\in M\left(\theta \right)$. The average number of iterations of the five decoding algorithms is $AI_{1}–AI_{6}$, while $d_{c}$ represents line weight and $d_{v}$ represents column weight. Table 1 gives the calculation methods of the total number of real operations of each decoding algorithm. From Table 1, it can be concluded that the complexity of the LUDWSF, LUDSMWSF and LUDMSMWSF algorithms is lower than the WSF, SMWSF and MSMWSF algorithms. Taking one algorithm as an example, the computation amount of WSF algorithm and LUDWSF algorithm is the same for each iteration with the difference of the average iteration times. However, the average iterations of LUDWSF algorithm are much less than that of WSF algorithm. From Table 1, the LUDMSMWSF algorithm only requires real additions, complexity is $Mq\left(2d_{c}-1\right)+Nqd_{v}+\left(N-1\right)+\left(N-1+d_{c}d_{v}\right)\left(AI_{5}-1\right)$. However, complexity of the Fast Fourier Transform-based belief propagation decoding algorithm (FFT-BP) [32,33] includes real additions, multiplications and divisions, the complexities of which are $AI_{6}\left[2Nd_{v}q\log_{2}q+2Nd_{v}\left(q-1\right)+M\left(d_{c}-1\right)\right]$, $AI_{6}\left[Nd_{v}q\left(d_{c}+2d_{v}-1\right)+Md_{c}\right]$ and $AI_{6}\left[Nd_{v}\left(q+2\right)\right]$, respectively. Real multiplications and divisions are more consumable units than real additions. Although the proposed WSF algorithm with flipping pattern requires more iterations for decoding than FFT-BP, it needs less real additions than FFT-BP and requires no multiplying the iterations with computational requirements in each iteration, the total computational requirement of WSF algorithm is still lower than FFT-BP. Therefore, the computational requirement of WSF algorithm is much lower than FFT-BP.

## 5. Simulation Results and Statistical Analysis

**Figure 2:**
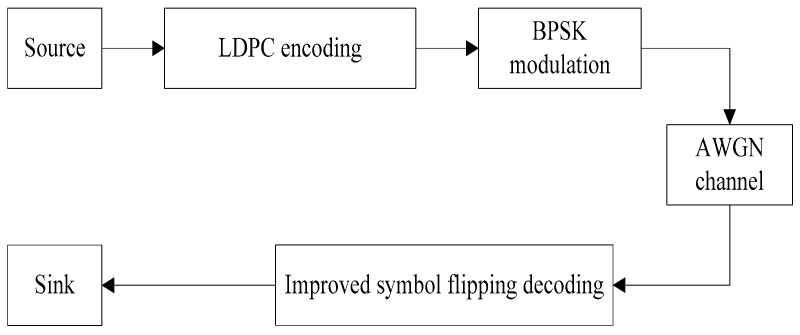
The link level simulation block diagram.

The simulation parameters used in this section are as follows: 384,192 LDPC codes with a code rate of 0.5 and a column weight of three. The matrix is generated by the progressive edge growth (PEG) algorithm [34,35], divided into 4-ary (Code 1) and 16-ary (Code 2) simulation, which has a maximum number of iterations of 100. Under the AWGN channel conditions and using BPSK modulation, at least 1000 error bits are collected at each SNR. The link level simulation block diagram is shown in Figure 2.

### 5.1. Weighted Factor Test

**Figure 3:**
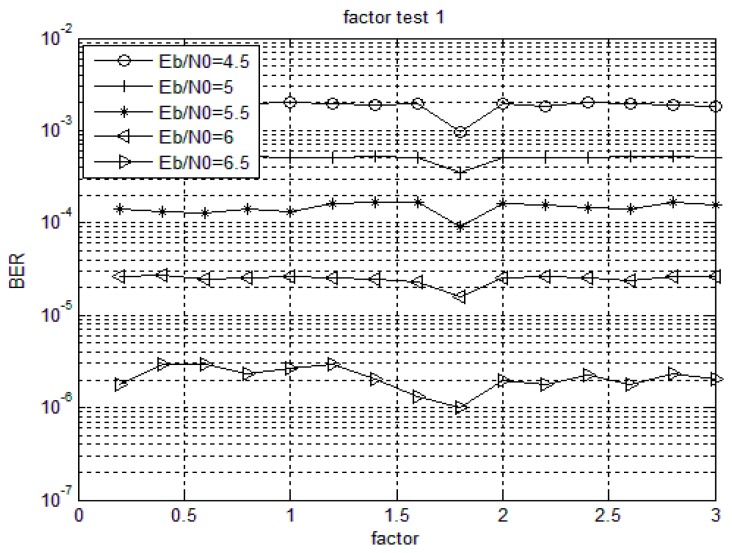
Code 1 weighted factor test.

**Figure 4:**
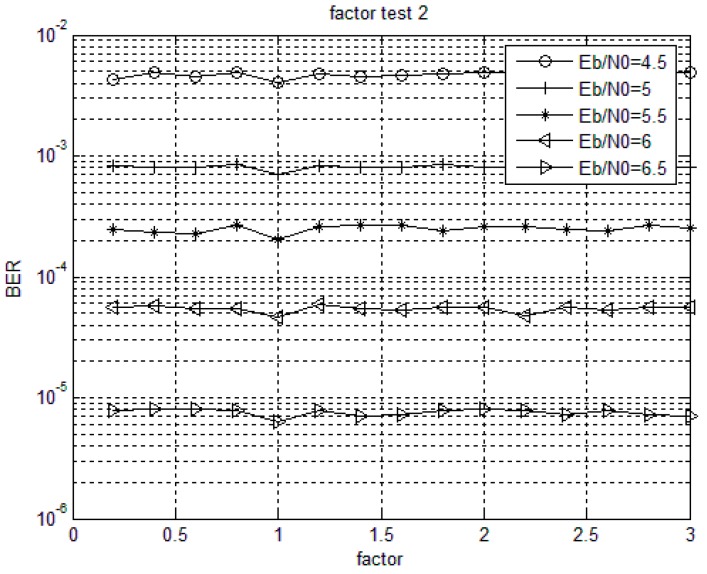
Code 2 weighted factor test.

For non-binary LDPC codes with given column weights, the decoding performance is different under the same signal-to-noise ratio with different weighting factors. When choosing a constant and optimal weighting factor, the performance loss is negligible and the complexity of the implementation is decreased [36,37]. The optimal value of the weighting factor is generally related to the non-binary LDPC codes and the specific code structure. Figure 3 and Figure 4 show the bit error rate performance of Code 1 and Code 2 with different weighting factors when using MSMWSF algorithm under different SNR conditions. Based on the definition of the optimal value of weighted factor, it can be seen from Figure 3 that the influence of weighting factor on bit error rate (BER) is not obvious at lower SNR as the optimal value of weighting factor varies little with an increase in SNR. As shown in Figure 3 and Figure 4, the optimal value of the weighting factor of the MSMWSF algorithm in Code 1 in this paper is 1.8, while the optimal value of the weighting factor of the MSMWSF algorithm in Code 2 is one.

### 5.2. Comparison of Algorithm Performance and Average Iteration Numbers

**Figure 5:**
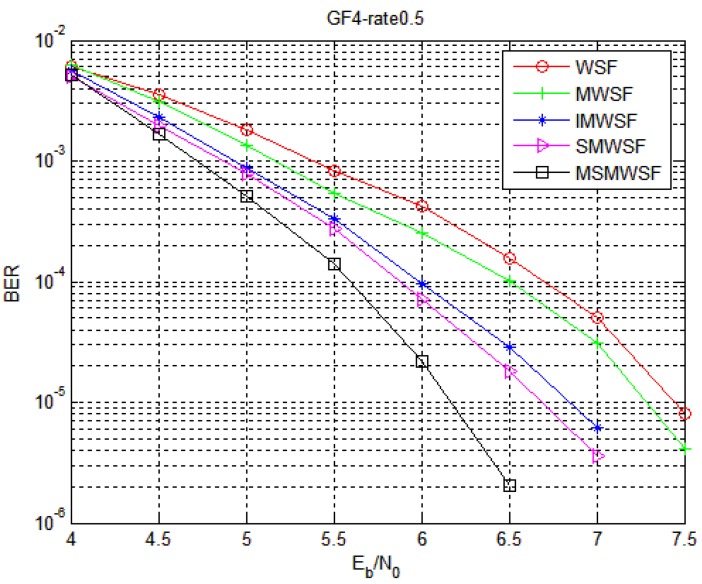
Comparison of five algorithms under Code 1.

**Figure 6:**
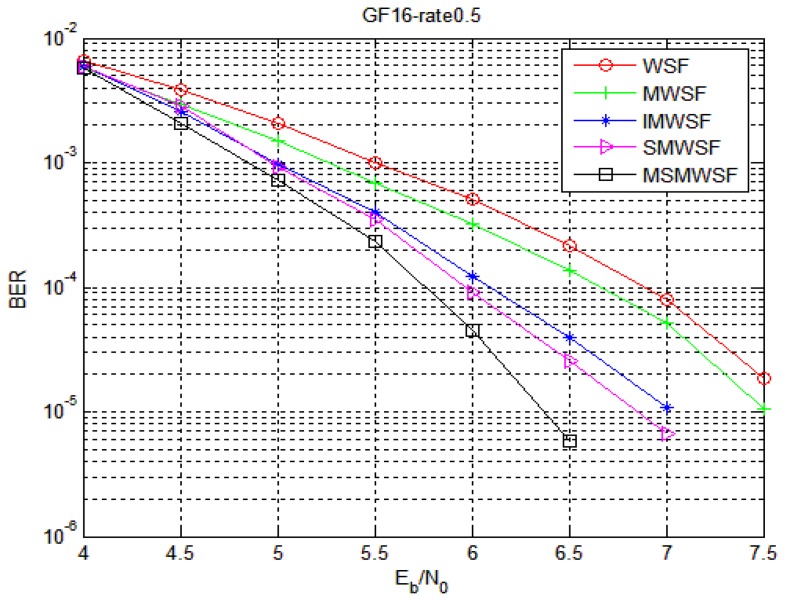
Comparison of five algorithms under Code 2.

The performance comparison between Code 1 and Code 2 in five different decoding algorithms under the optimal parameters is shown in Figure 5 and Figure 6, respectively. With an increase in SNR, the coding gain of MSMWSF algorithm is gradually increasing compared with other existing algorithms. At a low SNR, the performance of MSMWSF algorithm is almost the same as other algorithms. According to the simulation diagram of reference [34,35,36], a higher number of binary numbers indicates a poorer performance of the WSF algorithm. From Figure 5 and Figure 6, we can see that the performance of Code 1 is better than that of Code 2, which proves the effectiveness of the algorithm proposed in this paper.

**Figure 7:**
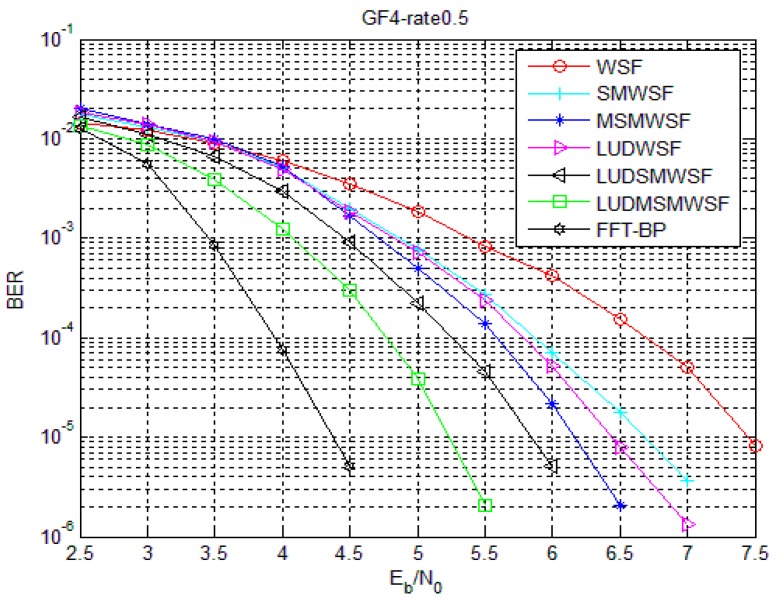
Comparison of improved algorithms under Code 1.

**Figure 8:**
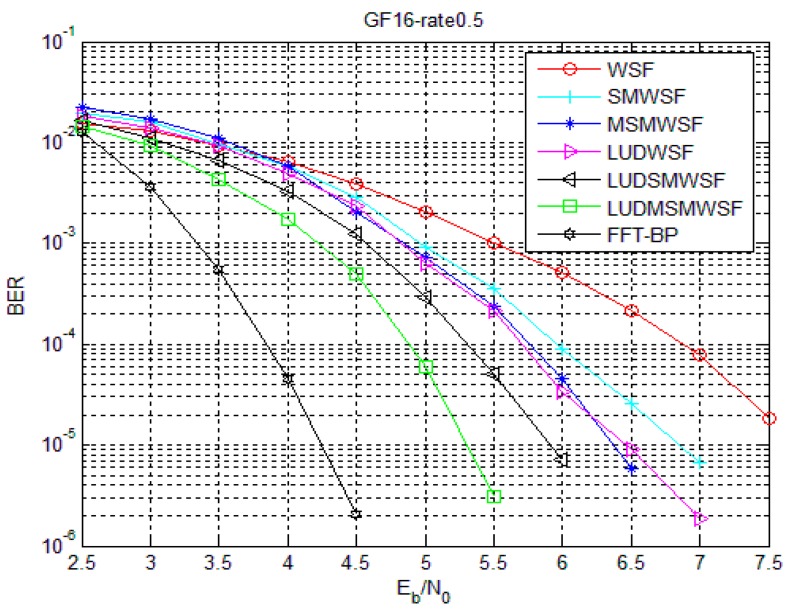
Comparison of improved algorithms under Code 2.

Figure 7 and Figure 8 show the performance of Code 1 and Code 2 under the SMWSF algorithm and MSMWSF algorithm, respectively, with the optimal parameters of the weighting factors after introducing the loop update detection algorithm. As seen from Figure 7 and Figure 8, the LUDMSMWSF algorithm improves the decoding performance compared to the MSMWSF algorithm, accelerating the convergence speed. Compared with the WSF algorithm, a coding gain of 2.2 dB is obtained in the case of Code 1 and a coding gain of 2.35 dB in the case of Code 2. Under the same decoding algorithm, the coding gain of about 0.8–1.1 dB can be obtained after introducing the proposed loop update detection algorithm. At the same time, the LUDMSMWSF algorithm is about 0.85–1.05 dB away from FFT-BP decoding at BER of 10^−5^ with a tremendous reduction of computational requirement. In view of the results on Figure 7 and Figure 8, we argue that the proposed symbol flipping algorithm offer the tradeoff points between performance and computational cost.

**Figure 9:**
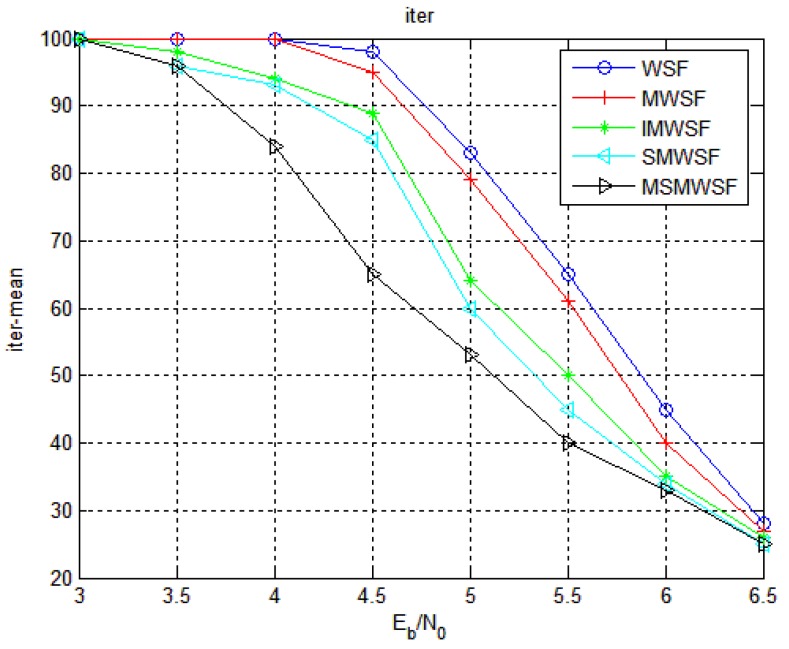
The average number of iterations of five algorithms under Code 1.

**Figure 10:**
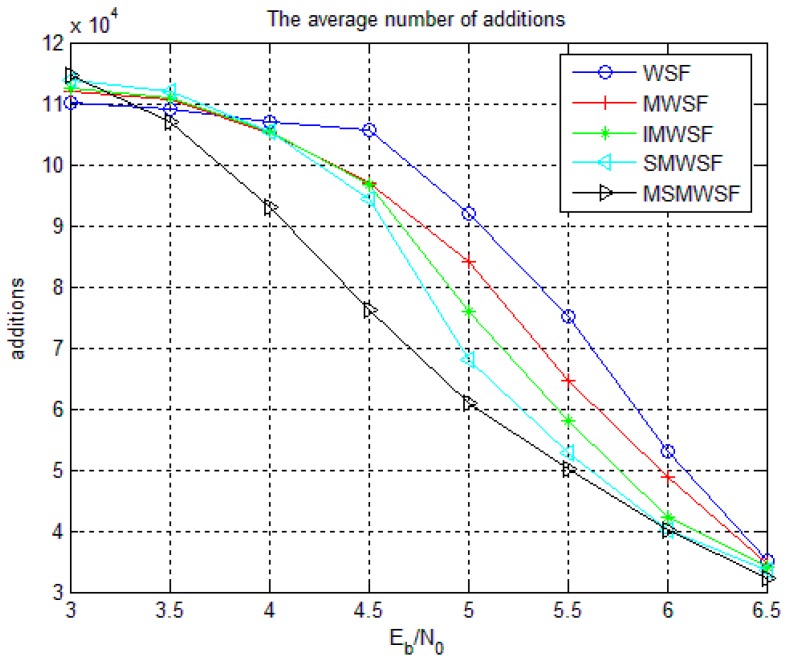
The average number of addition operations of five algorithms under Code 1.

Figure 9 and Figure 10 gives the average number of iterations and average number of addition operations of Code 1 under five decoding algorithms. As shown in Figure 9 and Figure 10, the two algorithms proposed in this paper are significantly less than the average number of iterations and the average number of addition operations in the traditional algorithm. Because the algorithm proposed in this paper has more efficient and accurate symbol flipping function, it improves the performance and reduces the complexity of the algorithm to a certain extent. We see that MSMWSF algorithm achieves fast convergence and low complexity.

We set the SNR to be 3.5 dB with the simulation of 10,000 frames under the condition of Code 1. We then combined the statistical five algorithms for decoding failure frames, as shown in Table 2. Table 2 shows that the WSF algorithm has nearly 99% frame failure. Two decoding algorithms are proposed in this paper. The failure of the frame is about 62% and 42%, which is less than traditional algorithms. It can also be seen that the algorithm proposed in this paper does not increase the complexity of the implementation, but has been reduced to a certain extent.

The total computation required for decoding this LDPC codewith three various algorithms at 4.5 dB is shown in Table 3. From Table 3, the FFT-BP algorithm is nearly six times the computational requirement of the MSMWSF algorithm only in real addition. Therefore, we can prove that the computational requirement of MSMWSF algorithm is much lower than FFT-BP with no real multiplication and division. The MSMWSF algorithm has the lowest complexity and does not need to consume hardware resources and multiplication and division operations in software overhead.

## 6. Conclusions

This paper proposes a sum of the magnitude for hard decision decoding algorithm based on loop update detection. The algorithm combines the magnitude of the sum information of the variable nodes adjacent to the check node and uses it as the reliability information. At the same time, the reliability information of the variable node itself is taken into account and a more effective flip function is obtained, which improves the flip efficiency of the symbol and improves the decoding performance of the algorithm. The loop update detection algorithm is introduced to improve the accuracy of symbol flipping and to further accelerate the convergence rate of decoding. Simulation results show that compared with the WSF algorithm, the proposed LUDMSMWSF algorithm gains about 1.3 dB and 1.8 dB respectively and the decoding complexity is greatly reduced. Therefore, the algorithm proposed in this paper can be better applied to the 5G mobile communication system and meet the requirements of the decoding algorithm. It is a good candidate decoding algorithm for high speed communication devices.

## Figures and Tables

**Table 1:** The average total number of real operations in each algorithm.

SF Algorithm	Addition Operations	Multiplication Operations	Division Operations
**WSF**	$\begin{array}{l} Mq\left(d_{c}-1\right)+Nq\left(d_{v}-1\right)+\left(N-1\right)\\ +\left(N-1+d_{c}d_{v}\right)\left(AI_{1}-1\right) \end{array}$	**0**	**0**
**SMWSF**	$\begin{array}{l} Mq\left(2d_{c}-1\right)+Nq\left(d_{v}-1\right)+\left(N-1\right)\\ +\left(N-1+d_{c}d_{v}\right)\left(AI_{2}-1\right) \end{array}$	**0**	**0**
**MSMWSF**	$\begin{array}{l} Mq\left(2d_{c}-1\right)+Nqd_{v}+\left(N-1\right)\\ +\left(N-1+d_{c}d_{v}\right)\left(AI_{3}-1\right) \end{array}$	**0**	**0**
**LUDSMWSF**	$\begin{array}{l} Mq\left(2d_{c}-1\right)+Nq\left(d_{v}-1\right)+\left(N-1\right)\\ +\left(N-1+d_{c}d_{v}\right)\left(AI_{4}-1\right) \end{array}$	0	0
**LUDMSMWSF**	$\begin{array}{l} Mq\left(2d_{c}-1\right)+Nqd_{v}+\left(N-1\right)\\ +\left(N-1+d_{c}d_{v}\right)\left(AI_{5}-1\right) \end{array}$	0	0
**FFT-BP**	$AI_{6}\left[\begin{array}{l} 2Nd_{v}q\log_{2}q\\ +2Nd_{v}\left(q-1\right)+M\left(d_{c}-1\right) \end{array}\right]$	$AI_{6}\left[\begin{array}{l} Nd_{v}q\left(d_{c}+2d_{v}-1\right)\\ +Md_{c} \end{array}\right]$	$AI_{6}\left[Nd_{v}\left(q+2\right)\right]$

**Table 2:** Five algorithms for decoding failure frames.

SF Algorithm	Decoding Failure Frames
WSF algorithm	9915
MWSF algorithm	9840
IMWSF algorithm	6544
SMWSF algorithm	6200
MSMWSF algorithm	4184

**Table 3:** Under the condition of Code 2, Eb/N0 = 4.5 dB, decoding complexity of three algorithms.

SF Algorithm	Addition Operations	Multiplication Operations	Division Operations
WSF algorithm	205644	0	0
MSMWSF algorithm	92710	0	0
FFT-BP algorihtm	567723	744870	66267
